# Comparative knowledge, attitudes, and practices regarding anthrax, brucellosis, and rabies in three districts of northern Tanzania

**DOI:** 10.1186/s12889-019-7900-0

**Published:** 2019-12-03

**Authors:** Christian Kiffner, Michelle Latzer, Ruby Vise, Hayley Benson, Elizabeth Hammon, John Kioko

**Affiliations:** 1 Center for Wildlife Management Studies, The School For Field Studies, PO Box 304, Karatu, Tanzania; 2 School of Public Health, College of Charleston, Charleston, SC 29401 USA; 3 Department of Integrative Biology, Oregon State University, Corvallis, OR 97331 USA; 4 College of Computer, Mathematics, and Natural Sciences, University of Maryland, College Park, MD 20742 USA; 5 School of Nursing, Clemson University, Clemson, SC 29632 USA

**Keywords:** Anthrax, Brucellosis, Knowledge, attitudes, and practices, Rabies, Risk perception, Zoonotic disease

## Abstract

**Background:**

Knowledge, attitudes, and practices (KAP) surveys regarding zoonotic diseases are crucial to understanding the extent of knowledge among citizens and for guiding health-related education programs.

**Method:**

Employing a structured questionnaire, we interviewed residents (*n* = 388) in three districts of northern Tanzania (Karatu *n* = 128, Monduli *n* = 114, Babati *n* = 146) to assess knowledge, attitudes and reported practices regarding three zoonotic diseases that occur in the region (anthrax, brucellosis, and rabies). We used generalized linear mixed effects models and multi-model inference to identify demographic correlates of knowledge.

**Results:**

Proportional average district- and disease- specific knowledge scores ranged from 0.14–0.61. We found positive correlations between age and knowledge of symptoms, causes and treatments of anthrax (three districts), brucellosis (three districts), and rabies (one district). Gender, ethnic identity, formal education and ownership of livestock or dogs had variable effects on knowledge among the interviewed population. Risk perceptions regarding different diseases varied across districts and were positively correlated with knowledge of the specific diseases. Direct interactions with livestock and domestic dogs were reported to occur across all demographic groups, suggesting that most people living in rural settings of our study area are potentially exposed to zoonotic diseases. Behaviors which may favor transmission of specific pathogens (such as consumption of raw milk or meat) were occasionally reported and varied by district. Wildlife was generally regarded as negative or neutral with regard to overall veterinary and human health.

**Conclusion:**

The combination of variable knowledge about zoonotic diseases in the three districts, reported occurrence of practices that are conducive to pathogen transmission, and previously documented circulation of pathogens causing anthrax, brucellosis and rabies in our study system, call for health education programs embedded in a holistic One Health approach.

## Background

Zoonotic diseases are of major concern for public health and impose a considerable burden on national and global economies [1, 2]. Compared to developed countries, veterinary and human health in developing countries are disproportionately affected by zoonotic pathogens because these countries are often located in the tropics and thus in areas of high pathogen species richness [3]. In addition, community dependence on livestock, high prevalence of bushmeat consumption, lack of food and water safety and security, and frequent interactions with wildlife may expose multiple sections of the human population to zoonotic pathogens [4–7]. Finally, a typically weak health infrastructure, insufficient training of medical and veterinary health workers, and inefficient cross-sectional collaboration between veterinarians, health practitioners and public health authorities frequently inhibit timely and appropriate diagnosis and treatment of zoonotic diseases [8, 9].

This general setting of potential exposure to zoonotic pathogens combined with limited medical diagnosis facilities and trained personnel in developing countries inevitably assigns substantial responsibility for disease prevention to individuals. Knowledge, attitude and practice studies (KAP) are suitable to assess the extent of knowledge among human populations and document current practices that possibly enhance risks for pathogen infections. Results from KAP studies are crucial to inform and guide public health education programs which attempt to close knowledge gaps and reduce the frequency of practices that potentially favor pathogen transmission [9–15]. Multiple demographic and other human related factors have been hypothesized to influence knowledge of a specific disease, and identifying particular subsets of the human population where knowledge is lacking, could greatly improve the efficacy of education programs [16]. For example, gender has been found to influence knowledge, with males often having greater knowledge about a specific disease [10]. Similarly, being potentially more exposed to a certain pathogen (e.g. living in an area with a high prevalence of a specific pathogen, or keeping animals that may be involved in the transmission of a specific pathogen), and having received formal education may also increase knowledge of a specific disease [10, 15, 17–19]. Beyond identifying knowledge gaps and documenting risky practices, KAP studies can further assess the general risk perception regarding different diseases among the local population, particularly if surveys target multiple diseases.

In this study we focused on three zoonotic diseases that are occasionally diagnosed from surveillance systems or research projects in humans living in rural northern Tanzania: anthrax, brucellosis, and rabies. Overall, these diseases may be considered ‘neglected’ diseases and their actual prevalence may be underreported in Tanzania due to challenges associated with integrated disease surveillance and response systems [20, 21], occasional unspecific symptoms of zoonotic diseases (e.g. for brucellosis), and misdiagnoses of zoonotic diseases by medical practitioners [9].

Anthrax (causative agent: *Bacillus anthracis*) outbreaks have been relatively well documented in our study area. Anthrax outbreaks have caused substantial declines in wild animal populations in the past [22] and the disease is diagnosed among residents of Monduli district [23] and the neighboring Serengeti-Ngorongoro ecosystem [24]. From 2013 to 2016, the reported incidence rate was 7.88 cases / 100,000 persons in the Arusha region of northern Tanzania [23]. Despite being eradicated in some parts of the world, brucellosis (causative agent: *Brucella* spp.) occurs in wildlife, livestock, and humans of northern Tanzania [9, 12, 19]. In northern Tanzania, *Brucella* seroprevalence of up to 7.7% in humans and 3–4.6% in livestock has been documented [19]. Rabies (causative agent: rabies virus) circulates in Tanzania (mainly in domestic dog populations but frequent cases are diagnosed among the human and wildlife populations) despite substantial vaccination efforts in several parts of the country [25–28]. Seroprevalence of rabies in unvaccinated dogs in northern Tanzania can reach 31.6% [29].

We employed a comparative approach to assess knowledge, attitudes and practices regarding these diseases in three districts of northern Tanzania. The study area differs in major land-use forms and contact rates with wildlife species, and captures a variety of ethnic/cultural backgrounds. The main objectives were to assess and compare (1) the knowledge and correlates of knowledge regarding these three diseases, (2) the prevalence of practices that potentially increase infection risk, and (3) risk perceptions towards the three diseases. Finally, (4) we investigated interviewees’ views towards wildlife with respect to veterinary and human health.

## Methods

### Study area

**Fig. 1:**
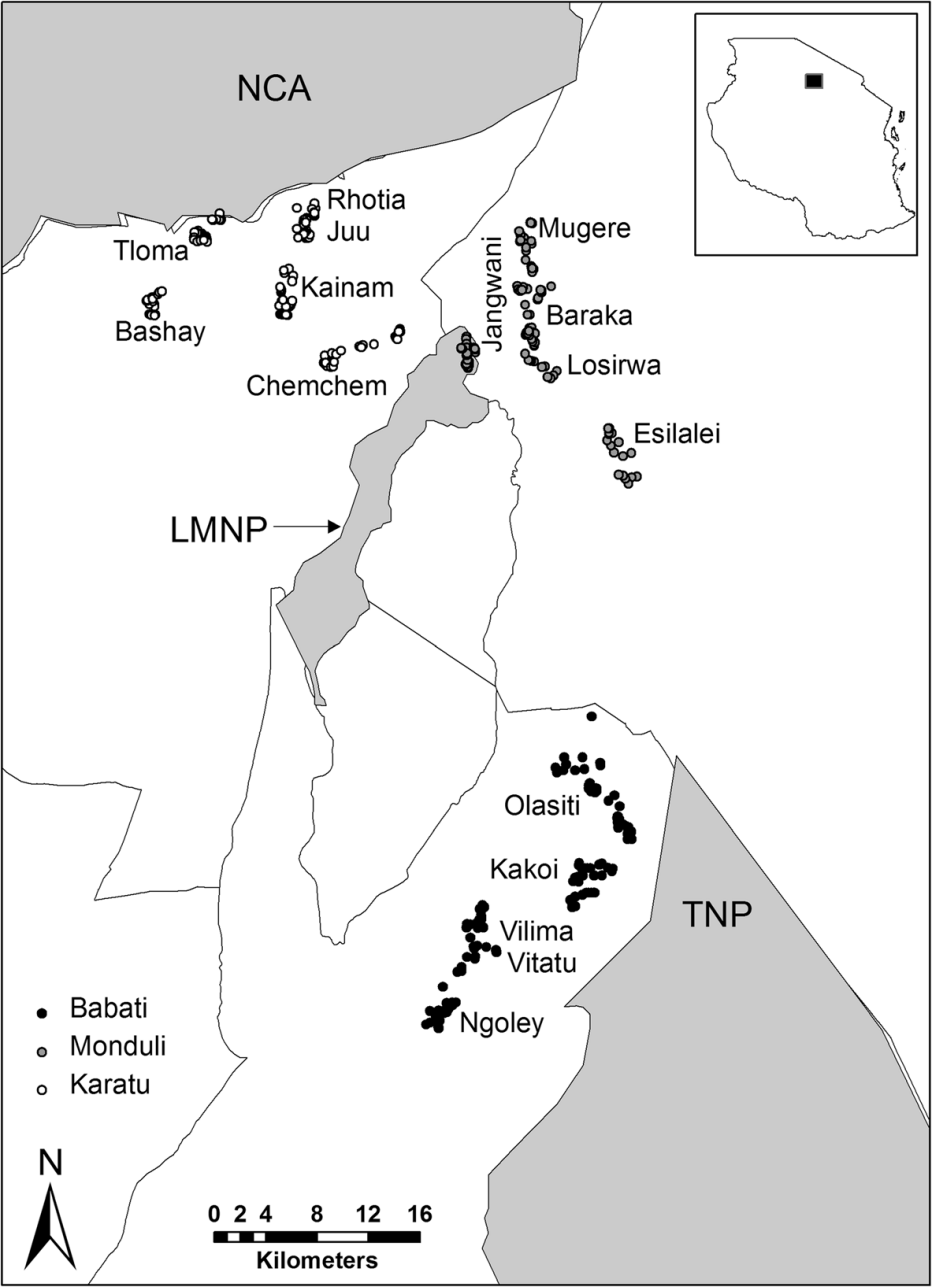
Map of study area. Locations of household interviews in relation to the major protected areas (NCA = Ngorongoro Conservation Area; LMNP = Lake Manyara National Park; TNP = Tarangire National Park), Lake Manyara (LM) and district boundaries. Households in the village ‘Jangwani’ are not inside LMNP but at its border; the impression that they may be inside the national park may be due to inaccuracies of the protected area shapefile. The inset on the top right indicates the approximate location of the study area within Tanzania. Shapefiles for protected areas and district boundaries are available at: https://protectedplanet.net/country/TZ and https://gadm.org/download_country_v3.html

This interview-based study was carried out in the Karatu, Monduli and Babati districts of northern Tanzania (Fig. 1). The Karatu district is mainly located in the Mbulu highlands [30], which are semi-arid to humid [31]. The Ngorongoro Conservation Area (NCA) and Lake Manyara National Park (LMNP) are bordering village lands in this district which brings several wildlife species near farms of the predominant Iraqw people in the area [30]. The Iraqw are mostly small-holder agriculturalists, cultivating the land with maize, beans, pigeon peas, barley and wheat [32]. The Monduli district is east of the Karatu district and is located in the lowlands of the Great Rift Valley [31]. This area is considered a semi-arid landscape [30] and contains several conservation areas with high densities of wildlife: Lake Manyara National Park (LMNP), Manyara Ranch Conservancy (MR), and the Mto wa Mbu Game-Controlled Area (GCA) [33, 34]. Within this district we mainly sampled people residing in the rural areas around Mto wa Mbu town, which are primarily inhabited by Maasai pastoralists [30]. Within the Babati district, the study took place in villages within the Burunge Wildlife Management Area (WMA). These villages are located close to Tarangire National Park (TNP) and Manyara Ranch Conservancy (MR). The villages are part of the Burunge Wildlife Management Area and are thus in proximity to areas dedicated to wildlife conservation. This part of the Babati district is a semi-arid area dominated by savannah habitats, and home to various ethnicities and agro-pastoral communities [30].

### Interviews

Within each of the three districts, we chose either five (Karatu and Monduli) or four (Babati) villages. We chose villages for their relatively even distribution across the districts, sufficient number of households and accessibility and willingness of village heads to support the study. We conducted interviews using a predetermined and pretested questionnaire over the course of ten days in April 2017 and four days in November 2017. Prior to field work, translators and investigators went through the interview questions to clarify the meaning of each question and translated the questions to Swahili. In addition, we conducted test interviews with residents of Rhotia. We recruited translators from the Cultural Tourism Program in Mto Wa Mbu; all of them had previous research experience with interview-based studies in our study areas. We conducted all interviews in Swahili and translators translated responses immediately to English and responses were recorded in English.

We conducted interviews along 3–5 transects in each village. Approximately every 100 m along each transect, a trained translator (accompanied by one or two investigators) asked a member of a homestead to voluntarily participate in the survey. We only conducted interviews with one person over the age of 18 per household, after gaining verbal consent from the participant. In total, we conducted 388 interviews (Karatu district *n* = 128; Monduli district *n* = 114; Babati district *n* = 146).

We first asked respondents if they were willing to take part in a 45-min survey on knowledge of zoonotic diseases in the area, prior to conducting the interview. We guaranteed participants anonymity as well as the right to stop the interview at any time, in accordance with protocols on rights of human subjects in research. Initially, we asked basic demographic information of the respondent (gender, age, highest level of education, ethnicity, and number of cattle/sheep&goats/dogs owned). We recorded ethnicity as either the predominant ethnicity for each district (i.e. the numerical majority in our sample) or “other” (Karatu: Iraqw vs. other, Monduli: Maasai vs. other, and Babati: Maasai and Arusha combined vs. other). Despite considered different ethnicities, Arusha and Maasai share a common language, a primarily pastoralist lifestyle and many other cultural similarities. We are aware that this may constitute an oversimplification of the ethnic background of interviewees. However, given the diversity of ethnic backgrounds in our study area, we felt that this approach aligned with our major goal (i.e. to identify major determinants of knowledge such as living a primarily pastoralist vs. a primarily farming lifestyle) while ensuring sufficient degrees of freedom in the models.

In line with previous KAP studies [10], we then asked questions that assessed the respondents’ knowledge regarding three zoonotic diseases: anthrax, brucellosis, and rabies. We first asked interviewees if they had heard about this disease. If interviewees had heard about the disease, we asked them whether this disease affect human and/or animals, what kind of symptoms may be present, how this disease can be transmitted, what kind of treatment should be used if a person or animal is infected, if infected persons should consult a medical doctor, and how transmission of this disease can be prevented (Additional file 1: Table S1).

After completing the interviews, responses were jointly assessed and transformed into knowledge points by the same two investigators against the stated criteria outlined in Table 1.

**Table 1:** Description of symptoms, causes, treatments, and prevention methods for anthrax, brucellosis, and rabies in humans

Disease	Symptoms	Causes	Treatment	Prevention
Anthrax	Small blisters or itchy bumps; Painless ulcer after blister; High fever/chills; Chest pain; Cough; Headache; Confusion/dizziness; Fatigue; Aches; Diarrhea; Stomach pain/swelling	Inhalation of dust from animal hide or hair Consumption of undercooked, infected meat; Skin to open wound contact	Seek medical care as quickly as possible; Antibiotics are routine treatment	Do not consume undercooked meat; Do not touch animal carcasses; Vaccines for humans and animals available
Anthrax scoring (max. of 10 points)	0 = 0 points 1–2 correct = 1 point 3 or above correct = 2 points Observed in humans or animals: None = 0 points Humans = 1 point Animals = 1 point Both = 2 points	0 = 0 points 1–2 correct = 1 point 3 or above correct = 2 points	0 = 0 points 1 correct = 1 point Seek professional help: Yes = 1 point No = 0 points	None = 0 points 1 correct = 1 point
Brucellosis	Fever of unknown origin; Nonspecific and assorted symptoms such as: sweats, weight loss, depression, arthralgias, fatigue, malaise; Can occur in any organ system of the body	Consuming unpasteurized milk or meat from infected animals; Direct contact with secretions of infected animals; Breathing in brucellosis bacteria	Seek immediate consultation from doctor; Antibiotic treatment regime	Boiling milk before consumption; Vaccination of livestock; Protection from dead animal/livestock tissue
Brucellosis scoring (max. of 10 points)	0 = 0 points 1–2 correct = 1 point 3 or above correct = 2 points Observed in humans or animals: None = 0 points Humans = 1 point Animals = 1 point Both = 2 points	0 = 0 points 1 correct = 1 point 2 or above correct = 2 points	0 = 0 points 1 correct = 1 point Seek professional help Yes = 1 point No = 0 points	None = 0 points 1 correct = 1 point 2 or above correct = 2 points
Rabies	Flu-like symptoms; Fever; Headache; Fatigue/weakness; Acute neurologic syndrome; Coma	Rabies Virus; In almost all cases virus is transmitted via bite of infected animal	Post exposure vaccines; Immune globulin	Vaccination of domestic pets; No direct contact/handling of wild animals
Rabies scoring (max. of 8 points)	0–1 = 0 points 2+ correct = 1 point Observed in Humans or Animals: None = 0 points Humans = 1 point Animals = 1 point Both = 2 points	Contact with infected animal = 1 point	Human: seek professional treatment = 1 point Animal: kill infected animal = 1 point	Vaccination or no contact with (infected/symptomatic) animals = 1 point

Below the row outlining symptoms, causes and prevention methods, we report the applied scoring system for each category. We assigned scores according to the provided answers and specific disease information provided by Centers For Disease Control and Prevention [35–37]

In addition to questions with regards to knowledge about the three diseases, we asked specific questions about practices that potentially enhance pathogen transmission and about demographic at-risk groups. For example, we asked which demographic groups in a household were responsible for livestock and dog handling. In addition, we asked interviewees about how they prepare or consume milk and meat because raw consumption of these animal products may increase pathogen infection risk (e.g. for brucellosis). Additionally, we asked interviewees whether the co-existence with wildlife generally increases, decreases or does not affect the health of livestock and people. At the end of the interview, we asked respondents to rank each disease in accordance to their perceived danger for human and livestock health; the lowest ranked disease embodied the most perceived danger.

### Data analyses

The relative knowledge of each disease was assessed based on the number of obtained knowledge points (i.e. points awarded to answers according to criteria in Table 1) relative to the maximum achievable points for the corresponding disease (anthrax: 10 points; brucellosis: 10 points; rabies: 8 points). ML and RV carefully and jointly read through all answers and assigned knowledge points according to the criteria provided in Table 1.

Because interviewees in the three districts differed considerably in terms of socio-demographic structure, we analyzed data separately for each district and disease. To identify which demographic variables (ethnicity, gender, age, level of education, livestock ownership, dog ownership) were associated with the level of knowledge, we used a generalized linear mixed model with binomial error distribution, using the *lme* package implemented in the software R [38, 39]. Because the level of knowledge was assessed as proportion (achieved knowledge points / maximum number of achievable points), analyzing the data with linear regression models is not appropriate [40]. To avoid introducing a subjective knowledge threshold, we specified our target variable “knowledge” for each disease as a two-column variable whereas the first column contains the “achieved” points (i.e. successes) and the second column the “missed” points (i.e. failures); columns for successes and failures were combined using the *cbind* function [40]. Therefore, our model estimates the influence of explanatory variables on the relative knowledge about a specific disease in a logistic regression framework. To account for clustering of interviews (i.e. responses from interviewees from one village may not entirely be independent), we included a random effect for each village. Prior to model fitting, pairwise correlations across explanatory variables were assessed using the *corrplot* package [41]; since none of the correlation exceeded the 0.7 collinearity threshold (Additional file 2: Figure S2), we included all variables in our models [42]. Due to the high number of a priori hypotheses (gender, age, level of education, ethnicity, livestock ownership for anthrax, and dog ownership for rabies) we first fitted a full model (including all hypothesized variables) and standardized regression coefficients (numeric variables with more than two values were rescaled to a mean of 0 and a standard deviation of 0.5; binary variables were rescaled to have a mean of 0 and a difference of 1 between their two categories) using the *arm* package [43]. We then ran all possible permutations of variable combinations (only using additive linear effects) using the *MumIn* package. Since multiple models had similar model selection support, we model averaged regression coefficients of models within Δ-AICc-values ≤6 using the full average method [44–46]. Model selection tables can be found in the electronic appendix (Additional file 3: Table S2). To predict model outcomes, we calculated odds ratios (exponent of regression coefficients) which describe the relative change in knowledge in response to the corresponding explanatory variable i.e., the relative change in knowledge compared to the reference level for categorical variables and the relative change in knowledge when continuous variables change by one unit (note that variables were standardized and the odds ratios thus relate to the mean of the explanatory variable). In line with information-theory we assessed variables based on relative variable importance (computed in the *MumIn* package) and confidence intervals of regression estimates.

To describe reported practices in relation to zoonotic disease infection risk and at-risk groups, we provide proportions of responses for each district. Proportions were based on the interview sample size in each district (Karatu district *n* = 128; Monduli district *n* = 114; Babati district *n* = 146); in case respondents answered multiple practices or at-risk groups, we created new categories for these answers. We used a Kruskal Wallis anova to test for significant differences in risk perception towards the three diseases and Kendall’s correlation test to assess associations between relative knowledge and risk perception of the three diseases.

## Results

### Socio-economic characteristics of the interviewees

We surveyed a total of 388 households in the districts of Karatu (*n* = 128 in five villages), Monduli (*n* = 114 in five villages), and Babati (*n* = 146 in four villages). In each village, we sampled between 17 and 40 households (Table 2). Overall, gender of respondents was relatively evenly distributed (Table 2). Proportionally, respondents in Karatu district were primarily of the Iraqw ethnicity (0.88), and were typically small-scale farmers. In Babati, and particularly in Monduli districts, a substantial share of the interviewees were Maasai (0.59 and 0.37, respectively) and lived a mainly pastoralist lifestyle (Table 2).

**Table 2:** Socio-demographic characteristics of interviewees in the three surveyed districts of northern Tanzania

	Karatu (#)	Karatu (prop.)		Monduli (#)	Monduli (prop.)		Babati (#)	Babati (prop.)
Households	128			114			146	
Gender								
Male	67	0.52		67	0.59		50	0.34
Female	61	0.48		47	0.41		96	0.66
	Mean	Range		Mean	Range		Mean	Range
Age	42	18–88		36	18–74		39	18–85
Ethnicity	(#)	(prop.)	Ethnicity	(#)	(prop.)	Ethnicity	(#)	(prop.)
Iraqw	112	0.88	Maasai	67	0.59	Maasai	54	0.37
Other	16	0.13	Other	47	0.41	Other	92	0.63
Education								
None	13	0.10		25	0.22		25	0.17
Primary	88	0.69		61	0.54		80	0.55
Secondary and above	27	0.21		28	0.25		41	0.28
Livestock ownership								
Yes	100	0.78		80	0.70		112	0.77
No	28	0.22		34	0.30		34	0.23
	Mean	Range		Mean	Range		Mean	Range
Number of cattle	3	0–50		20	0–320		13	0–200
Number of goats & sheep	4	0–35		38	0–400		20	0–300
Dog ownership	(#)	(prop.)		(#)	(prop.)		(#)	(prop.)
Yes	73	057		71	0.62		95	0.65
No	55	0.43		43	0.38		51	0.35
Villages	Households	(prop.)	Villages	Households	(prop.)	Villages	Households	(prop)
Bashay	21	0.16	Baraka	17	0.15	Kakoi	33	0.23
Chem Chem	26	0.20	Esilalei	17	0.15	Ngoley	40	0.27
Rhotia Juu	29	0.23	Jangwani	29	0.25	Olasiti	36	0.25
Rhotia Kainam	27	0.21	Losirwa	28	0.25	Vilima Vitatu	37	0.25
Tloma	25	0.20	Mugere	23	0.20			

Frequencies for each category are given in absolute (#) and proportional (prop.) terms

Primary education was most common in each district, followed by secondary (and above) education but a substantial fraction of the interviewees (0.10–0.20 of respondents) had not received any formal education. In terms of dog and livestock ownership, interviewees in the three districts appeared similar, but owing to the predominant pastoralists’ ethnicities in Monduli and Babati, interviewees in these two districts usually had larger livestock herds compared to people residing in Karatu district (Table 2).

### Knowledge about zoonotic diseases

**Fig. 2:**
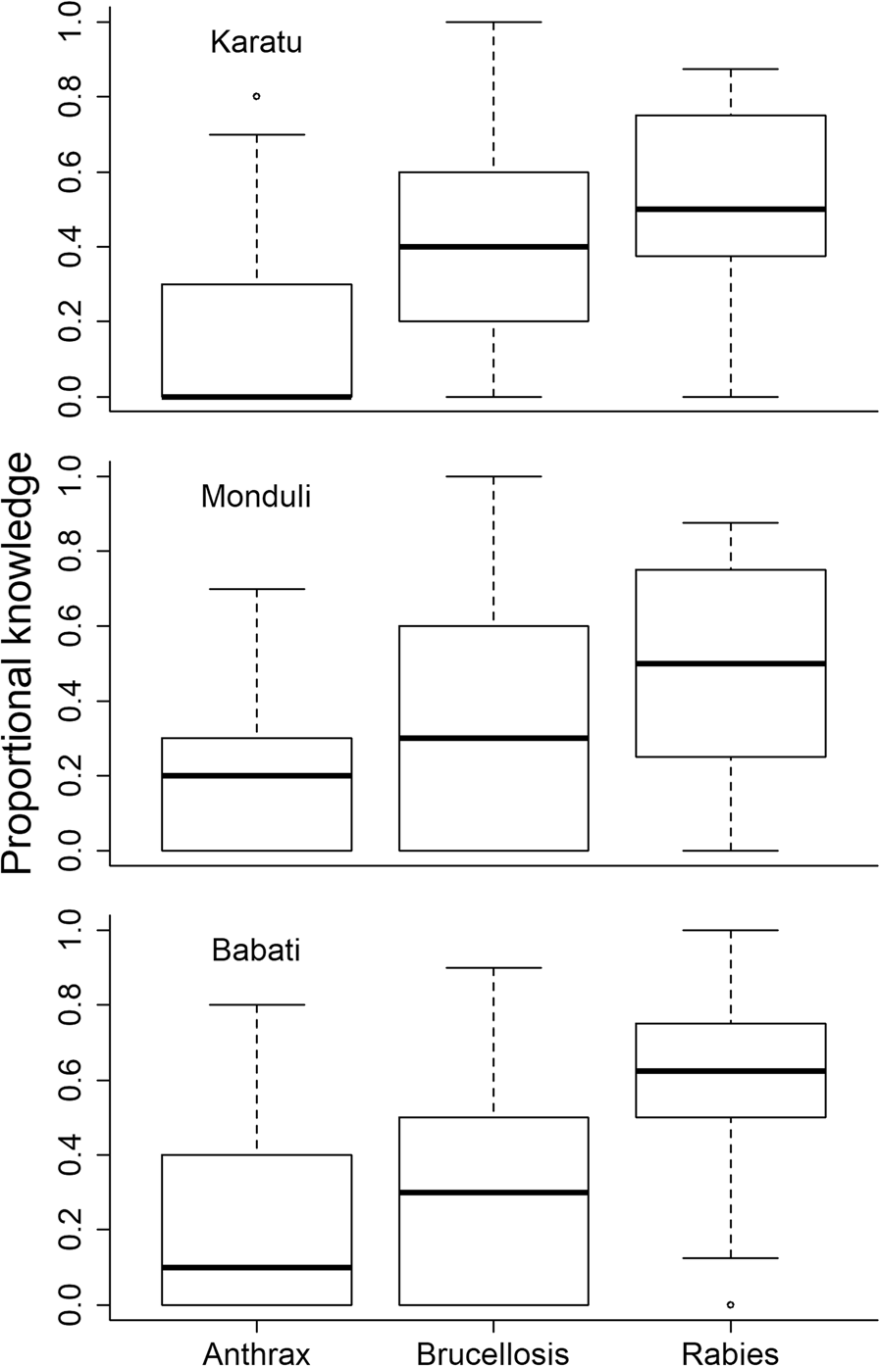
Proportional knowledge of zoonotic diseases among local residents in the districts of Karatu, Monduli and Babati in northern Tanzania. The midline represents the median, and the upper and lower limits of the box denote third and first quartile, respectively. Whiskers indicate the range and open circles represent outlier

Knowledge regarding anthrax, brucellosis, and rabies varied across districts (Fig. 2). In Karatu, respondents were most informed about rabies, followed by brucellosis, and least informed about anthrax. Interviewees in the district of Monduli also had greater knowledge of brucellosis, and rabies compared to anthrax. Respondents in Babati had greater knowledge of rabies, and anthrax than other districts, but comparatively little knowledge on brucellosis (Fig. 2).

According to generalized linear mixed models and subsequent model selection, age [based on relative variable importance (RVI) and confidence intervals non-overlapping with zero] was the major influential factor affecting anthrax knowledge in all three districts (Table 3). Regarding anthrax, the odds of scoring more points increased by 2.02–2.61 times per year of life (Table 3; please note that age was centered; i.e. the average age was scaled to zero). Gender was found to influence anthrax knowledge in both Karatu and Babati districts. In males the odds for scoring more knowledge points were 1.89–2.28 greater than for females. In Karatu, Iraqw respondents had less knowledge of anthrax than other ethnicities residing in the area. Formal education was positively associated with knowledge about anthrax, but confidence intervals of the regression coefficients overlapped with zero which implies that this relationship was not consistent or very strong (Table 3).

**Table 3:** Model averaged regression coefficients (β) incl. Associated 95%-confidence intervals (upper; lower), odds ratio (OR) and relative importance (RVI) of variables related to knowledge of anthrax, brucellosis and rabies in three districts of northern Tanzania

	Karatu			Monduli			Babati		
	β	OR	RVI	β	OR	RVI	β	OR	RVI
Anthrax									
Intercept	**−2.72 (−3.64;1.80)**			**−1.86 (−2.37;-1.34)**			**−1.66 (−2.13; −1.18)**		
Ethnicity (other vs. predominant)	**0.84 (0.36; 1.31)**	**2.31**	**1.00**	−0.19 (−0.72; 0.34)	0.83	0.49	0.44 (−0.09; 0.96)	1.55	0.84
Gender (m vs. f)	**0.83 (0.44; 1.21)**	**2.28**	**1.00**	0.21(−0.18; 0.61)	1.24	0.68	**0.64 (0.35; 0.93)**	**1.89**	**1.00**
Livestock ownership (yes vs. no)	0.16 (− 0.30; 0.63)	1.18	0.48	0.13 (− 0.28; 0.54)	1.14	0.45	− 0.02 (− 0.20; 0.16)	0.98	0.26
Education (primary vs. none)	0.63 (−0.15; 1.41)	1.88	0.88	0.34 (−0.12;0.81)	1.41	0.89	0.21 (−0.26; 0.69)	1.23	0.62
Education (secondary vs. none)	1.07 (−0.04; 2.19)	2.92	0.88	**0.64 (0.00; 1.29)**	**1.90**	**0.89**	0.35 (−0.31; 1.01)	1.42	0.62
Age	**0.96 (0.47; 1.45)**	**2.61**	**1.00**	**0.86 (0.55; 1.18)**	**2.37**	**1.00**	**0.71 (0.40; 1.01)**	**2.02**	**1.00**
Stdv. intercept	0.53			0.30			0.06		
Brucellosis									
Intercept	**−0.26 (−0.53; 0.00)**			**−0.70 (−0.94; − 0.46)**			**−1.19 (−1.76; − 0.63)**		
Ethnicity (other vs. predominant)	0.01(−0.17; 0.19)	1.01	0.25	**0.45 (0.02; 0.87)**	**1.56**	**0.93**	**0.76 (0.31; 1.21)**	**2.14**	**1.00**
Gender (m vs. f)	0.01 (−0.12; 0.15)	1.01	0.26	−0.02 (− 0.16; 0.13)	0.98	0.25	0.01 (−0.13; 0.16)	1.01	0.27
Livestock ownership (yes vs. no)	−0.12 (− 0.42; 0.18)	0.89	0.54	−0.03 (− 0.24; 0.19)	0.97	0.28	− 0.11 (− 0.40; 0.18)	0.90	0.50
Education (primary vs. none)	0.01 (−0.19; 0.20)	1.01	0.20	0.02 (−0.13; 0.16)	1.02	0.11	0.11 (−0.27; 0.49)	1.11	0.33
Education (secondary vs. none)	0.05 (−0.25; 0.35)	1.05	0.20	0.01(−0.14;0.16)	1.01	0.11	0.11 (−0.30; 0.52)	1.12	0.33
Age	**0.46 (0.21;0.71)**	**1.59**	**1.00**	**0.41 (0.15; 0.66)**	**1.50**	**1.00**	**0.53 (0.27; 0.80)**	**1.70**	**1.00**
Stdv. intercept	0.19			0.18			0.46		
Rabies									
Intercept	0.19 (−0.19; 0.56)			**−0.64 (−1.12; −0.17)**			**0.44 (0.23; 0.65)**		
Ethnicity (other vs. predominant)	−0.02 (− 0.25; 0.21)	0.98	0.27	0.12 (−0.33; 0.57)	1.13	0.39	0.12 (−0.20; 0.45)	1.13	0.49
Gender (m vs. f)	**0.56 (0.30; 0.83)**	**1.76**	**1.00**	0.28 (−0.08;0.63)	1.32	0.83	0.02 (−0.13; 0.18)	1.02	0.28
Dog ownership (yes vs. no)	0.07 (−0.16; 0.31)	1.08	0.43	0.14 (−0.23; 0.51)	1.15	0.52	−0.32 (− 0.65; 0.01)	0.73	0.91
Education (primary vs. none)	−0.03 (− 0.40; 0.33)	0.97	0.51	**0.65 (0.27; 1.03)**	**1.92**	**1.00**	0.02 (−0.15; 0.19)	1.02	0.16
Education (secondary vs. none)	−0.22 (− 0.83; 0.39)	0.80	0.51	**0.69 (0.25; 1.14)**	**2.00**	**1.00**	0.04 (−0.19; 0.27)	1.04	0.16
Age	**0.51 (0.17; 0.86)**	**1.67**	**0.99**	0.01 (−0.14; 0.16)	1.01	0.25	0.04 (−0.14; 0.22)	1.04	0.33
Stdv. intercept	0			0.34			0.05		

Regression coefficient estimates are averages of models within 6 AICc scores of the most supported models. Estimates with confidence intervals non-overlapping with zero are highlighted in bold. The standard deviation of the intercept denotes variability in the random intercept among the five (Karatu and Monduli) and four (Babati) villages

Knowledge pertaining to brucellosis was positively associated with age of respondents in all three districts. For the districts Monduli and Babati, ethnicity was also found to be an important determinant of knowledge, with members of other ethnicities knowing relatively more about rabies compared to interviewees of Maasai (Monduli) or Maasai and Arusha (Babati) ethnic identity (Table 3).

In Karatu district, gender explained some differences in knowledge about rabies. In male respondents, the odds for scoring greater knowledge points were 1.76 times greater compared to female interviewees. In this district, age positively influenced respondent’s knowledge as well. Among respondents in Monduli, interviewees with a primary education or secondary education had about two times greater odds to score more knowledge points compared to interviewees without formal education. In the Babati district, none of the regression estimates were strongly associated with knowledge of rabies (Table 3).

### Practices and risk factors related to zoonotic diseases

**Fig. 3:**
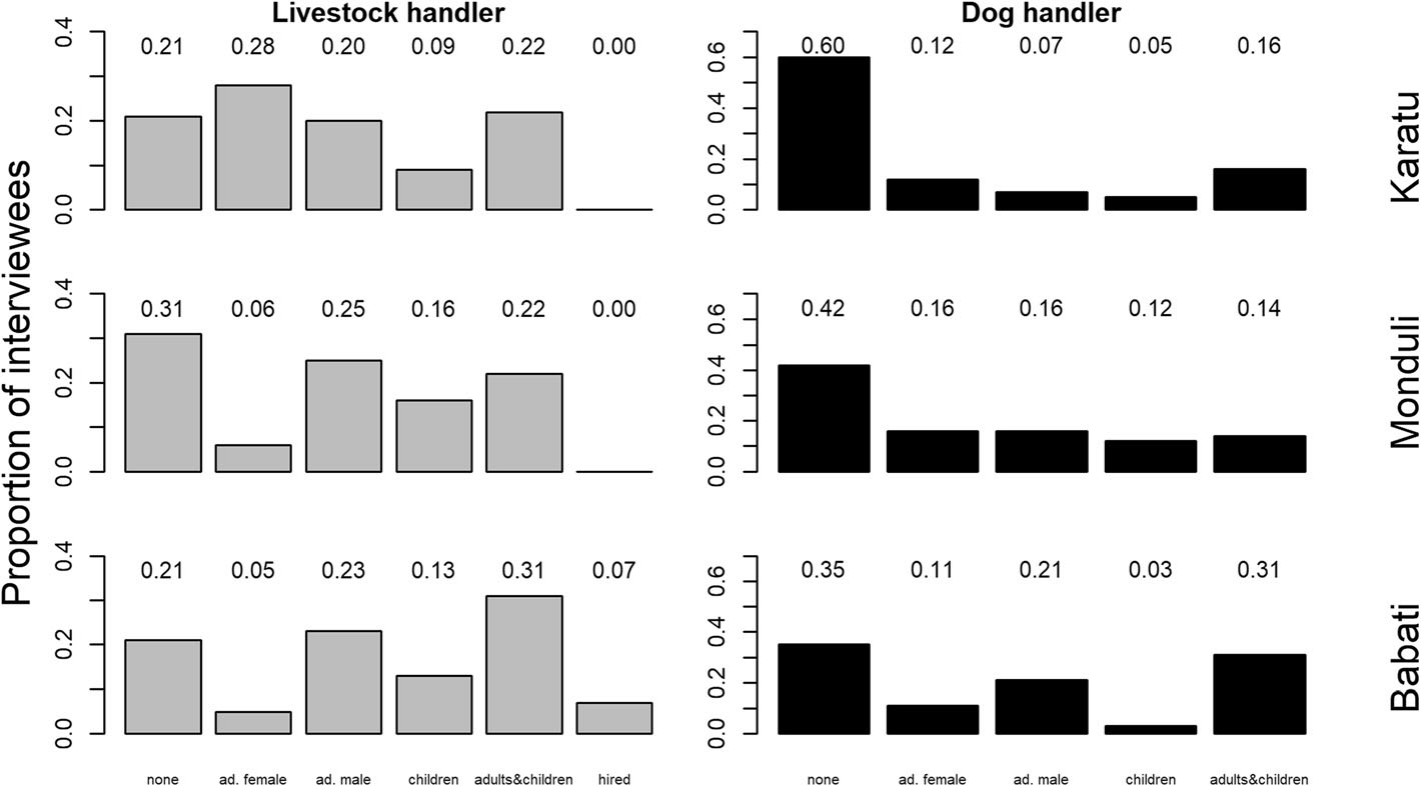
Reported proportion of demographic groups (ad. = adult) responsible for livestock and dog handling among interviewed households in three districts (Karatu, Monduli, and Babati) of northern Tanzania. Scores denote the exact proportion of each bar category

Overall, all demographic groups within a household reportedly took care of livestock and dogs (Fig. 3) but the main demographic group differed across districts. In Karatu, adult females were often mainly responsible for livestock (0.28 of respondents), whereas in Monduli (0.06) and Babati (0.05), adult females were rarely solely handling livestock. Children were also reported (adults and children combined: 0.31–0.44) to handle livestock in all districts. Similarly, dog handling was reportedly carried out by all demographic groups, including children (Fig. 3); children were major dog handler (children and adults & children combined) in a fifth to a third (0.21–0.36) of all households. Dog ownership in Monduli and Babati was also more prevalent compared to Karatu (Fig. 3; Table 2).

**Fig. 4:**
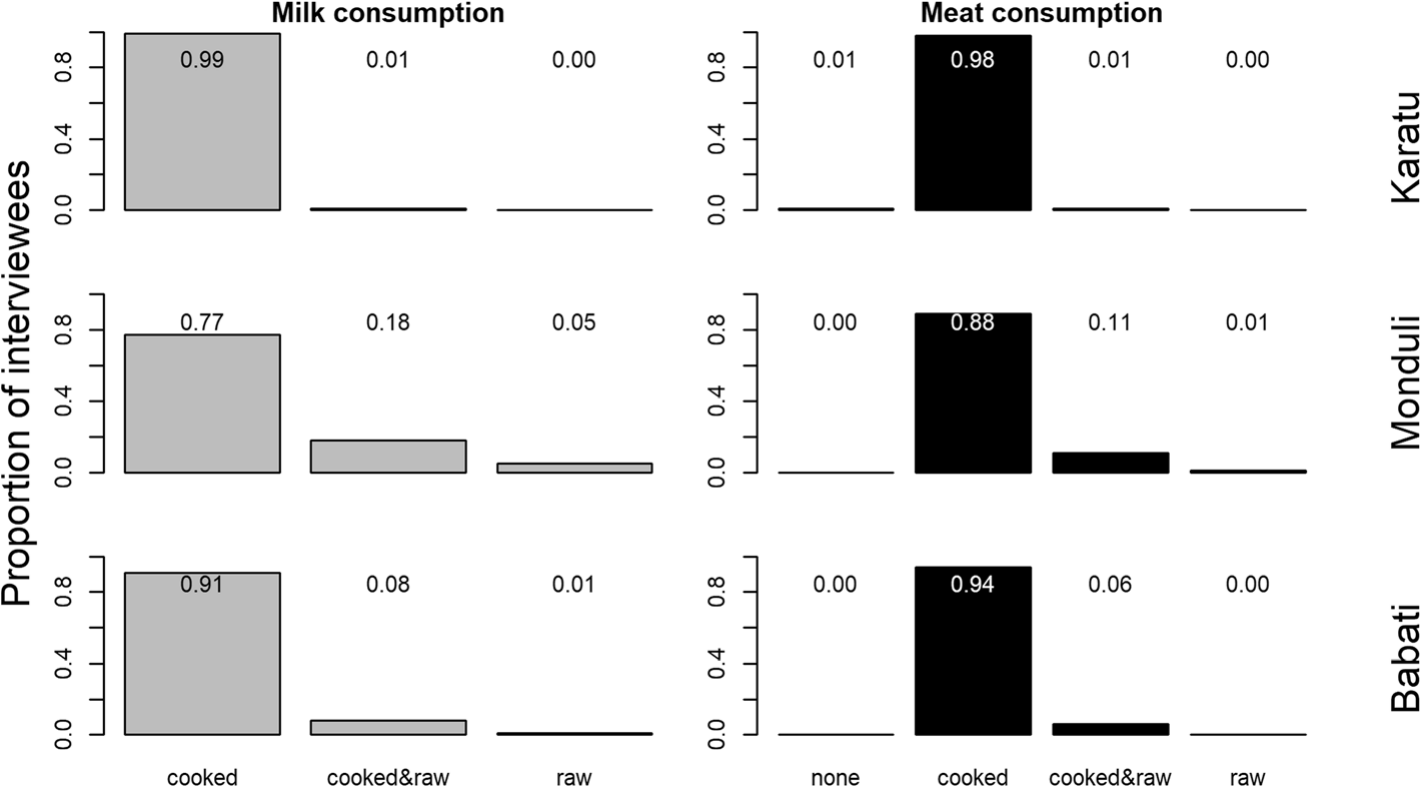
Reported preparation of milk and meat before consumption as reported by households in three districts (Karatu, Monduli, and Babati) of northern Tanzania. Scores denote the exact proportion of each bar category

The majority of interviewees in all three districts reported that milk (Karatu: 0.99; Monduli 0.77; Babati: 0.91) and meat (Karatu: 0.98; Monduli 0.88; Babati:0.94) were always boiled or cooked before consumption (Fig. 4). However, particularly in the districts of Monduli and Babati, a notable proportion of respondents reported consuming raw milk (Karatu: 0.01; Monduli 0.23; Babati: 0.09) and raw meat (Karatu: 0.01; Monduli 0.12; Babati: 0.06).

### Attitudes towards zoonotic diseases

**Fig. 5:**
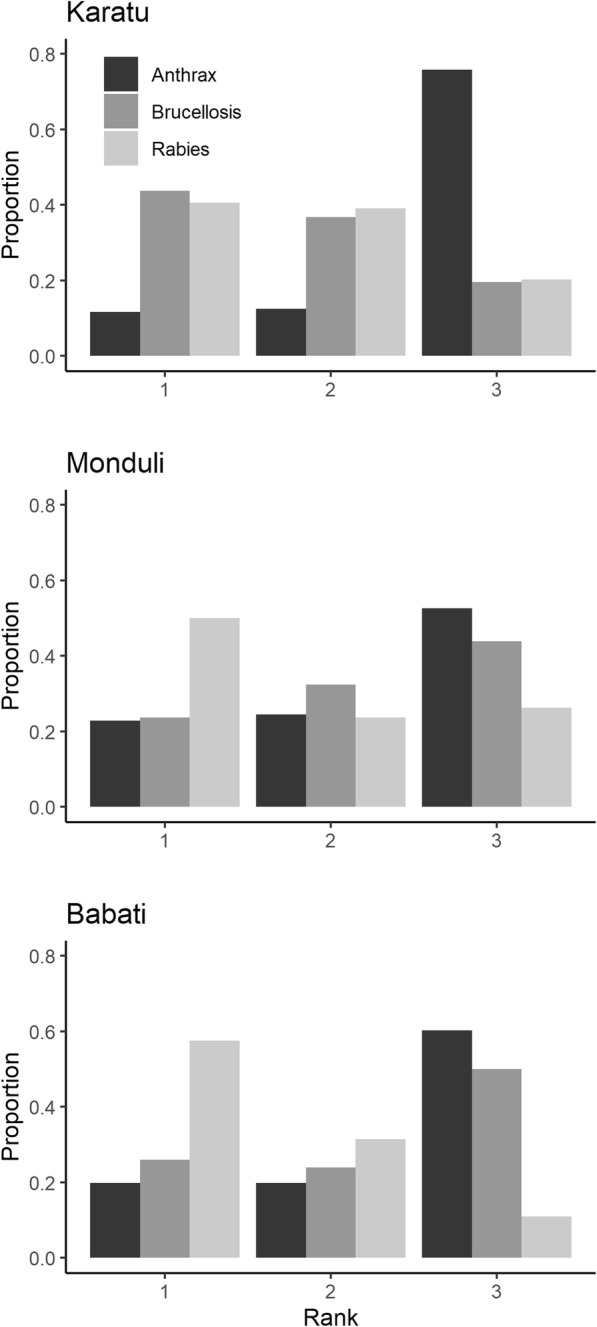
Local peoples’ risk perceptions of anthrax, brucellosis, and rabies in three districts (Karatu, Monduli, Babati) of northern Tanzania. For each district, we plotted the proportion of the disease rank by dividing the frequency of occurrence of each rank by the total number of respondents (Karatu *n* = 128, Monduli *n* = 114, Babati *n* = 146). Rank 1 displays highest risk perception, rank 3 lowest risk perceptions

Interviewees in Monduli and Babati districts frequently ranked rabies as the most dangerous disease, whereas interviewees in Karatu district rated brucellosis (closely followed by rabies) as a major threat to human and livestock health. Anthrax was consistently rated as least dangerous disease in all three districts (Fig. 5). Within all three districts, rankings of the three diseases were significantly different (Kruskal Wallis Χ^2^ > 25; df = 2; *p* < 0.001 for comparisons within districts). In all three districts, risk ranks were significantly (all *p* ≤ 0.001) correlated with the proportional knowledge of each disease (Karatu: tau = − 0.38, *n* = 384; Monduli: tau = − 0.36, *n* = 342; Babati: tau = − 0.39, *n* = 438). Given our risk scale (1 = most dangerous; 3 = least dangerous), people with greater knowledge about a specific disease tended to perceive it as more dangerous.

### Attitudes towards wildlife in relation to zoonotic diseases

**Fig. 6:**
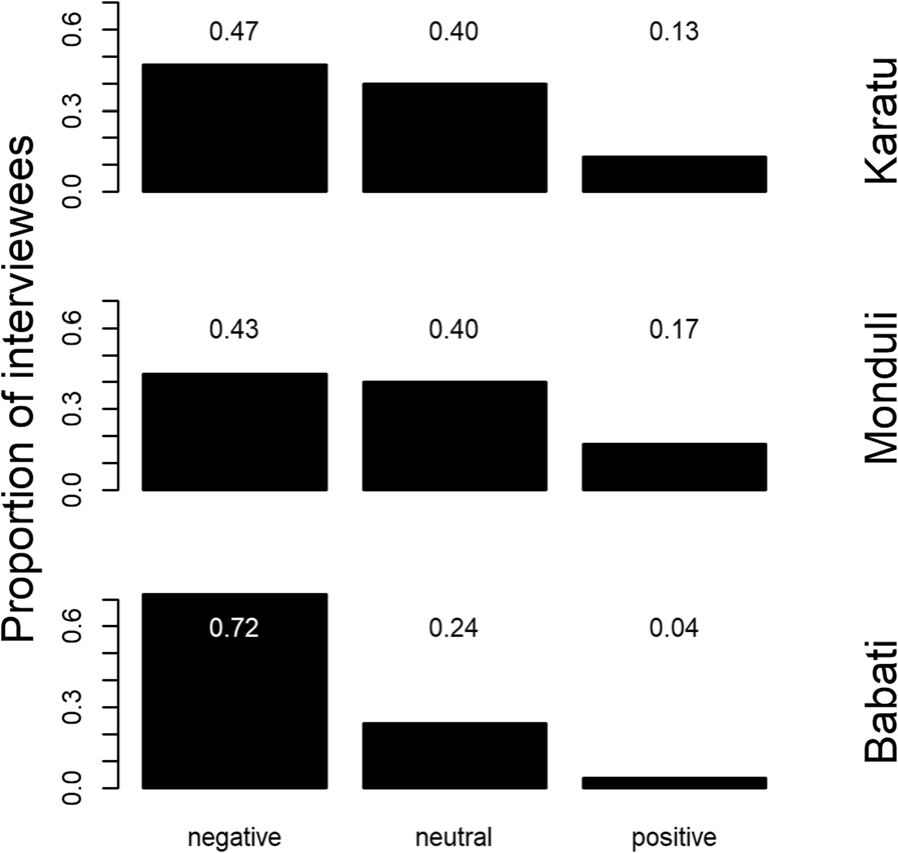
Proportion of local respondents’ attitudes (neutral, positive, negative) about the overall effect of wildlife on livestock and human health in three districts (Karatu, Monduli, and Babati) of northern Tanzania.

In all three districts, a large share of local people (Karatu: 0.47; Monduli: 0.43; Babati 0.72) expressed that wildlife had an overall negative influence on human and livestock health (Fig. 6). However, large proportions of the interviewees, particularly in Karatu and Monduli, mentioned that wildlife had a neutral (Karatu: 0.40; Monduli: 0.40; Babati: 0.24) or even a positive effect (Karatu: 0.13; Monduli: 0.17; Babati: 0.04) on human and livestock health.

## Discussion

Our KAP survey across three districts of northern Tanzania revealed that (1) relative knowledge about causes, symptoms, initial treatments and prevention methods regarding three zoonotic diseases (anthrax, brucellosis, and rabies) was very variable. Interestingly, (2) knowledge about specific diseases varied across districts, and (3) socio-demographic correlates of knowledge were rather variable in direction and strength with the exception of the frequently observed positive association between interviewee’s age and knowledge regarding knowledge of anthrax (all three districts), brucellosis (all three districts), and rabies (Karatu district). In addition, our survey revealed that (4) practices such as drinking raw milk and eating raw meat are practiced among a sizeable portion (reported proportional raw consumption in the three districts ranged from 0.01–0.23 for milk and 0.01 to 0.12 for meat) of the surveyed population, particularly in the districts with a greater share of ethnicities practicing pastoralist lifestyles. Finally, we show that (5) risk perceptions of diseases were positively and relatively consistently correlated with knowledge regarding specific diseases in each district.

### Knowledge and practices related to zoonotic diseases

Limited level of knowledge regarding zoonotic diseases, both among the rural population [10] and among medical practitioners [9], and limited access to treatments or post exposure prophylaxis [47], constitute favorable conditions for zoonotic pathogen transmission. Beyond appropriate public health laws, education, and individual behavior can possibly minimize the transmission of infectious diseases. Identifying knowledge gaps in human populations can thus be helpful [48, 49]. KAP studies are useful for identifying such knowledge gaps across landscapes and different demographic groups of the public, yet are associated with some level of uncertainty and potential bias. Our comparative approach required standardization in the interview process and the consistent scoring of answers according to pre-defined criteria. The trade-off for this standardization may be that we have underestimated actual knowledge due to possible language barriers (e.g. Swahili may not be the first language for all interviewees) and little efforts by interviewers to prompt more answers.

Despite these possible limitations, interviewees knew comparatively little about anthrax, with slightly greater knowledge scores achieved in the districts of Monduli and Babati (Table 3). Greater knowledge in Monduli and Babati can possibly be explained by historic anthrax outbreaks in Lake Manyara National Park (which is located in Monduli district, and borders Babati district) during the 1970s and 1980s [22, 50]. Importantly, Monduli district is still considered a high risk area for anthrax, with multiple instances of mainly cutaneous anthrax cases diagnosed in health facilities in this district [23]. During the dry season of 2018 an anthrax outbreak occurred in Babati district, mainly at the eastern shore of Lake Manyara, with numerous verified anthrax cases in wildebeest *Connochaetes taurinus* and other wildlife species (C. Laizer, pers. comm). In Monduli, and possibly also in Babati, the consumption of bushmeat is relatively common across large fractions of local residents [51], which may be a risk factor for exposure to anthrax given the high prevalence of *B. anthracis* in samples of wildlife species that are usually consumed by humans [23]. Indeed, anthrax infection risk seems to be particularly high in demographic groups that are frequently involved in handling animals (milking, slaughtering, skinning) [52].

Similar to other studies [10, 53], the findings of age being a key determinant of knowledge and spatially heterogeneous distribution of knowledge, provides some circumstantial evidence that knowledge about specific diseases is possibly affected by experience of past disease outbreaks and interventions in a given area. However, this hypothesis is difficult to test given the scarcity of reliable and comparative data on actual disease prevalence in space and time. Alternatively, the effect of age may merely indicate greater accumulated knowledge over a person’s lifespan (e.g. greater likelihood of exposure to public health information campaigns). Regardless of the underlying reasons for age being positively correlated with increased knowledge, this relationship supports the need for increased health education covering zoonoses among younger generations.

Seemingly, greater potential exposure to a zoonotic disease did not necessarily equate to greater knowledge in other cases. For example, the primarily pastoralist ethnicities in Monduli and Babati knew less about brucellosis compared to other non-primarily pastoralist ethnicities (Table 3). However, these relationships may also have been blurred by actual differences in brucellosis prevalence and possibly also by language barriers.

Formal education only had limited effects on knowledge regarding zoonotic diseases. An exception may be Monduli district, where education was positively associated with knowledge for anthrax and rabies. However, given the correlative nature of our study, it is not possible to accredit greater knowledge to specific education regarding zoonotic diseases in schools per se. To our knowledge, neither primary nor O-levels school syllabi in Tanzania explicitly cover aspects of the three diseases. Similarly, we are not aware of specific information campaigns on these diseases in our study area. Yet, before employing information campaigns in the school system or outside of formal education systems, it may be instrumental to formally assess the effectiveness of these education programs.

Because other hypothesized predictors were inconsistently related to knowledge regarding the three diseases and knowledge was occasionally lower in high risk groups compared to people possibly not as exposed, we suggest that a proactive educating system in the frame of a holistic One Health approach should be implemented in our study area [52]. Such education should particularly target individuals with elevated exposure to zoonotic diseases including children who often handle livestock and dogs in our study area and are thus exposed to potential infection. To some extent, variable exposure to specific pathogens reflects differences in cultural practices, and ethnic-specific gender roles [19, 52], which can be used to better identify specific risk groups. In light of frequent interactions between children, livestock and dogs (and thus potential exposure of children to associated zoonotic pathogens), it may be worthwhile to consider including basic aspects of zoonotic risk prevention in primary school education.

### Attitudes towards zoonotic diseases and risk perception

Unfortunately, there is only limited information (actual exposure risk and associated morbidity) about the relative importance of each disease in terms of public health in the region which prevents us to ascertain an objective disease risk ranking. In addition, the question was asked in a general sense which could have caused variable interpretation of the question among interviewees. Yet, considering a near 100% fatality rate in humans (if no post-exposure prophylaxis is administered), rabies may objectively be the most dangerous of the considered diseases [28, 35]. Indeed, a great proportion of respondents in Monduli and Babati rated rabies as the most dangerous disease (Fig. 5). Rabies prevalence is often underestimated in northern Tanzania [28, 54] and incidences may substantially be underreported. Apart from small-scale vaccination trials in Babati district [55], we are not aware of large scale dog (and cat) vaccination projects in the three studied districts. In light of the considerable effectiveness of mass vaccinations of domestic dogs and cats in reducing rabies [25, 27], this option should be jointly considered by public health authorities, local communities and international organizations [56] .

### Attitudes towards wildlife in relation to zoonotic diseases

Our interview data (Fig. 6) largely reflect overall negative perceptions regarding wildlife in this study area [57] and suggest that – with regard to human and veterinary health – the majority of rural people mainly perceive costs associated with wildlife, and few people consider wildlife species as beneficial for human and veterinarian health. Beyond the potential for pathogen transmission, large wildlife species cause direct and indirect costs to rural population in northern Tanzania [57]. Therefore, beyond improved education on zoonotic diseases, integrated “One Health” efforts need to strengthen the veterinary and medical infrastructure (hospitals and veterinary offices; reasonable transport options to health facilities; appropriate diagnosis tools and methods), services (e.g. preventive vaccination programs; affordable or free consultation at hospitals and veterinary district offices), and effective co-operation across the human health, veterinary, and wildlife sector. In turn, such investments in public health may additionally improve wildlife conservation efforts since improvements in veterinary health services could be associated with reductions in the severity of human-wildlife conflicts, because healthier livestock are possibly less prone to attacks by large carnivores [58].

## Conclusion

This KAP survey covering three diseases and spanning three districts highlights substantial knowledge gaps among the rural population in northern Tanzania. Proactively educating rural populations (i.e. before the occurrence of disease outbreaks), particularly targeting ethnic and demographic groups with elevated exposure risk to specific pathogens, could be a valuable tool to minimize transmission of zoonotic pathogens. Although clear evidence that education effectively reduces infection risk is often lacking [59], we hypothesize that education could be cost-effective method to reduce infection risk.

## Supplementary information

**Additional file 1: Table S1.** Questionnaire used to assess knowledge, attitudes and perceptions regarding anthrax, brucellosis, and rabies among residents of three districts in northern Tanzania. The questionnaire is provided in English and Swahili (*SW*).

**Additional file 2: Figure S2.** Pairwise Spearman rank correlation coefficients across explanatory variables. The variable “Dog ownership” (Dogs) was only used in the rabies model, whereas livestock ownership (Livestock) was used in anthrax, and brucellosis models.

**Additional file 3: Table S2.** Model selection tables for logistic regression models explaining variation in knowledge about anthrax, brucellosis, and rabies in three districts (Karatu, Monduli, Babati) of northern Tanzania. For each disease-district combination we conducted separate analyses.

## Data Availability

Data are publicly available and can be accessed at: https://datadryad.org/stash/share/K_rGX39KB03BtFztEp9xUjUQ42kKFyzwLcydqxI8Ntc.

## Abbreviations

Ad: Adult
AICc: sample size corrected Akaike information criterion
GCA: Mto wa Mbu Game-Controlled Area
KAP: Knowledge, attitudes, and practices
LM: Lake Manyara
LMNP: Lake Manyara National Park
MR: Manyara Ranch Conservancy
NCA: Ngorongoro Conservation Area
TNP: Tarangire National Park

## References

[1] Binder S, Levitt AM, Sacks JJ, Hughes JM (1999). Emerging infectious diseases: public health issues for the 21st century. Science (80- ).

[2] Morens DM, Folkers GK, Fauci AS (2004). The challenge of emerging and re-emerging infectious diseases. Nature..

[3] Guernier V, Hochberg ME, Guégan J-F (2004). Ecology drives the worldwide distribution of human diseases. PLoS Biol.

[4] Barua M, Bhaqwat SA, Jadhav S (2012). The hidden dimensions of human–wildlife conflict: health impacts, opportunity and transaction costs. Biol Conserv.

[5] Clifford DL, Kazwala RR, Sadiki H, Roug A, Muse EA, Coppolillo PC (2013). Tuberculosis infection in wildlife from the Ruaha ecosystem Tanzania: implications for wildlife, domestic animals, and human health. Epidemiol Infect.

[6] Wilkie D (2006). Bushmeat: a disease risk worth taking to put food on the table?. Anim Conserv.

[7] Wolfe ND, Daszak P, Kilpatrick AM, Burke DS (2005). Bushmeat hunting, deforestation, and prediction of zoonotic disease emergence. Emerg Infect Dis.

[8] Mbugi EV, Kayunze KA, Katale BZ, Kendall S, Good L, Kibik GS (2012). ‘One health’ infectious diseases surveillance in Tanzania: are we all on board the same flight?. Onderstepoort J Vet Res.

[9] John K, Kazwala R, Mfinanga GS (2008). Knowledge of causes, clinical features and diagnosis of common zoonoses among medical practitioners in Tanzania. BMC Infect Dis.

[10] Sambo M, Lembo T, Cleaveland S, Ferguson HM, Sikana L, Simon C (2014). Knowledge, attitudes and practices (KAP) about rabies prevention and control: a community survey in Tanzania. PLoS Negl Trop Dis.

[11] Mangesho PE, Neselle MO, Karimuribo ED, Mlangwa E, Queenan K, Mboera LEG (2017). Exploring local knowledge and perceptions on zoonoses among pastoralists in northern and eastern Tanzania. PLoS Negl Trop Dis.

[12] Ntirandekura J, Matemba LE, Ngowi HA, Kimera SI, Karimuribo ED (2018). Knowledge, perceptions and practices regarding brucellosis in pastoral communities of Kagera region in Tanzania. J Adv Vet Anim Res.

[13] Kioko J, Baker J, Shannon A, Kiffner C (2015). Ethnoecological knowledge of ticks and treatment of tick-borne diseases among Maasai people in northern Tanzania. Vet World.

[14] Stull JW, Peregrine AS, Sargeant JM, Weese JS (2012). Household knowledge , attitudes and practices related to pet contact and associated zoonoses in Ontario, Canada. BMC Public Health.

[15] Mfinanga SG, Mørkve O, Kazwala RR, Cleaveland S, Sharp JM, Shirima G (2003). The role of livestock keeping in tuberculosis trends in Arusha, Tanzania. Int J Tuberc Lung Dis.

[16] Krentel A, Fischer P, Manoempil P, Supali T, Servais G, Rückert P (2006). Using knowledge, attitudes and practice (KAP) surveys on lymphatic filariasis to prepare a health promotion campaign for mass drug administration in Alor District, Indonesia. Trop Med Int Heal.

[17] Yimer E, Mesfin A, Beyene M, Bekele A, Taye G, Zewdie B (2012). Study on knowledge, attitude and dog ownership patterns related to rabies prevention and control in Addis Ababa, Ethiopia. Ethiop Vet J.

[18] Dzikwi AA, Ibrahim AS, Umoh JU (2012). Knowledge and practice about rabies among children receiving formal and informal education in Samaru, Zaria, Nigeria. Glob J Health Sci.

[19] John K, Fitzpatrick J, French N, Kazwala R, Kambarage D, Mfinanga GS (2010). Quantifying risk factors for human brucellosis in rural northern Tanzania. PLoS One.

[20] Mghamba J, Mboera L, Krekamoo W, Senkoro K, Rumisha S, Shayo E (2004). Challenges of implementing an integrated disease surveillance and response strategy using the current health management information system in Tanzania. Tanzan J Health Res.

[21] CDC. Tanzania Surveillance (GHSA) in Action [Internet]. 2019 [cited 2019 Sep 19]. Available from: https://www.cdc.gov/globalhealth/security/stories/tanzania-surveillance-ghsa-in-action.html

[22] Prins HHT, Weyerhaeuser FJ (1987). Epidemics in populations of wild ruminants: anthrax and impala, rinderpest and buffalo in Lake Manyara National Park, Tanzania. Oikos.

[23] Mwakapeje ER, Høgset S, Fyumagwa R, Nonga HE, Mdegela RH, Skjerve E (2018). Anthrax outbreaks in the humans - livestock and wildlife interface areas of northern Tanzania: a retrospective record review 2006-2016. BMC Public Health.

[24] Hampson K, Lembo T, Bessell P, Auty H, Packer C, Halliday J (2011). Predictability of anthrax infection in the Serengeti, Tanzania. J Appl Ecol.

[25] Hampson K, Dushoff J, Cleaveland S, Haydon DT, Kaare M, Packer C (2009). Transmission dynamics and prospects for the elimination of canine rabies. PLoS Biol.

[26] Gascoyne SC, Laurenson MK, Lelo S, Borner M (1993). Rabies in African wild dogs (Lycaon pictus) in the Serengeti region, Tanzania. J Wildl Dis.

[27] Fitzpatrick MC, Hampson K, Cleaveland S, Meyers LA, Townsend JP, Galvani AP (2012). Potential for rabies control through dog vaccination in wildlife-abundant communities of Tanzania. PLoS One.

[28] Cleaveland S, Fe EM, Kaare M, Coleman PG (2002). Estimating human rabies mortality in the United Republic of Tanzania from dog bite injuries. Bull World Health Organ.

[29] Mtui-Malamsha N, Sallu R, Mahiti GR, Mohamed H, OleNeselle M, Rubegwa B (2019). Ecological and epidemiological findings associated with zoonotic rabies outbreaks and control in Moshi, Tanzania, 2017–2018. Int J Environ Res Public Health.

[30] Prins HHT (1987). Nature conservation as an integral part of optimal land use in East Africa: the case of the Masai ecosystem of northern Tanzania. Biol Conserv.

[31] Loth PE, Prins HHT (1986). Spatial patterns of the landscape and vegetation of Lake Manyara National Park. ITC J.

[32] Lawi YQ (1999). Where physical and ideological landscapes meet: landscape use and ecological knowledge in Irawq, northern Tanzania, 1920s-1950s. Int J Afr Hist Stud.

[33] Kiffner C, Nagar S, Kollmar C, Kioko J (2016). Wildlife species richness and densities in wildlife corridors of northern Tanzania. J Nat Conserv.

[34] Kiffner C, Rheault H, Miller E, Scheetz T, Enriquez V, Swafford R (2017). Long-term population dynamics in a multi-species assemblage of large herbivores in East Africa. Ecosphere..

[35] Centers For Disease Control and Prevention. Rabies [Internet]. 2017 [cited 2018 Feb 15]. Available from: https://www.cdc.gov/rabies/

[36] Centers For Disease Control and Prevention. Anthrax [Internet]. 2017 [cited 2018 Feb 15]. Available from: https://www.cdc.gov/anthrax/index.html

[37] Centers For Disease Control and Prevention. Brucellosis [Internet]. 2017 [cited 2018 Feb 15]. Available from: https://www.cdc.gov/brucellosis/index.html

[38] R Core Team. R: a language and environment for statistical computing [internet]. Vienna: R Foundation for statistical Computing; 2016. Available from: http://www.r-project.org/

[39] Bates D, Maechler M, Bolker B, Walker S. lme4: Linear mixed-effects models using Eigen and S4 [Internet]. 2015 [cited 2015 Oct 20]. Available from: http://cran.r-project.org/package=lme4

[40] Crawley MJ (2005). Statistics: an introduction using R.

[41] Wei T, Simko V. R package “corrplot”: Visualization of a Correlation Matrix (Version 0.84) [Internet]. 2017 [cited 2018 Jun 4]. Available from: https://github.com/taiyun/corrplot

[42] Dormann CF, Elith J, Bacher S, Buchmann C, Carl G, Carré G (2013). Collinearity: a review of methods to deal with it and a simulation study evaluating their performance. Ecography (Cop).

[43] Gelman A, Su Y-S. Arm: data analysis using regression and multilevel/hierarchical models. [Internet]. 2016 [cited 2018 Feb 15]. Available from: https://www.rdocumentation.org/packages/arm/versions/1.9-3

[44] Bárton K. Model selection and model averaging based on information criteria (AICc and alike) [Internet]. 2013 [cited 2017 Dec 23]. Available from: https://cran.r-project.org/web/packages/MuMIn/index.html

[45] Grueber CE, Nakagawa S, Laws RJ, Jamieson IG (2011). Multimodel inference in ecology and evolution: challenges and solutions. J Evol Biol.

[46] Richards SA (2008). Dealing with overdispersed count data in applied ecology. J Appl Ecol.

[47] Changalucha Joel, Steenson Rachel, Grieve Eleanor, Cleaveland Sarah, Lembo Tiziana, Lushasi Kennedy, Mchau Geofrey, Mtema Zacharia, Sambo Maganga, Nanai Alphoncina, Govella Nicodem J., Dilip Angel, Sikana Lwitiko, Ventura Francesco, Hampson Katie (2019). The need to improve access to rabies post-exposure vaccines: Lessons from Tanzania. Vaccine.

[48] Regidor E, De Mateo S, Calle ME, Domínguez V (2002). Educational level and mortality from infectious diseases. J Epidemiol Community Health.

[49] Magnusson R (2017). Controlling the spread of infectious diseases. Advancing the right to health: the vital role of law.

[50] Prins HHT, van der Jeugd HP (1993). Herbivore population crashes and woodland structure in East Africa. J Ecol.

[51] Kiffner C, Peters L, Stroming A, Kioko J (2015). Bushmeat consumption in the Tarangire-Manyara, Tanzania. Trop Conserv Sci.

[52] Mwakapeje ER, Høgset S, Softic A, Mghamba J, Nonga HE, Mdegela H (2018). Risk factors for human cutaneous anthrax outbreaks in the hotspot districts of northern Tanzania: an unmatched case – control study. R Soc Open Sci.

[53] Kovacic V, Tirados I, Esterhuizen J, Mangwiro CTN (2016). We Remember . Elders ’ Memories and Perceptions of Sleeping Sickness Control Interventions in West Nile. Uganda PLoS Negl Trop Dis.

[54] World Health Organization. The Control of Neglected Zoonotic Diseases: A route to poverty alleviation [Internet]. 2005 [cited 2018 Oct 9]. p. 54. Available from: http://www.who.int/zoonoses/Report_Sept06.pdf

[55] Lankester FJ, Wouters PAWM, Czupryna A, Palmer GH, Mzimbiri I, Cleaveland S (2016). Thermotolerance of an inactivated rabies vaccine for dogs. Vaccine..

[56] Bardosh K, Sambo M, Sikana L, Hampson K, Welburn SC (2014). Eliminating rabies in Tanzania? Local understandings and responses to mass dog vaccination in Kilombero and Ulanga districts. PLoS Negl Trop Dis.

[57] Bencin H, Kioko J, Kiffner C (2016). Local people’s perceptions of wildlife species in two distinct landscapes of northern Tanzania. J Nat Conserv.

[58] Khorozyan I, Soofi M, Khaleghi Hamidi A, Ghoddousi A, Waltert M (2015). Dissatisfaction with veterinary services is associated with leopard (Panthera pardus) predation on domestic animals. PLoS One.

[59] Ward DJ (2011). The role of education in the prevention and control of infection: a review of the literature. Nurse Educ Today.

