# Supplementary material for: Comparative knowledge, attitudes, and practices regarding anthrax, brucellosis, and rabies in three districts of northern Tanzania
Source: BMC Public Health. 2019 Dec 3;19:1625. doi: 10.1186/s12889-019-7900-0 (PMC6889212; doi:10.1186/s12889-019-7900-0)
Supplement: Supplementary file 1 — Additional file 1: Table S1. Questionnaire used to assess knowledge, attitudes and perceptions regarding anthrax, brucellosis, and rabies among residents of three districts in northern Tanzania. The questionnaire is provided in English and Swahili (SW). [file 12889_2019_7900_MOESM1_ESM.docx]

| Interviewer name  *SW:* ***Unaitwa nani?*** |  | Village  *SW:* ***Kijiji*** |  | Easting  *SW:* ***Masahriki*** |  | Southing  *SW:* ***Kusini*** |  | Age  *SW:* ***Una miaka mingapi?*** |  |
| --- | --- | --- | --- | --- | --- | --- | --- | --- | --- |
| Gender  *SW:* ***Jinsia yako?*** |  | Religion *SW:* ***Wewe ni dini gani?*** |  | Highest Education  *SW:* ***Kiwango cha elimu***  ***/***  ***Umesoma hadi darasa la ngapI?*** |  | Ethnicity  *SW:* ***Wewe ni kabila gani?*** |  | # in household  *SW:* ***Kwenye kaya yako mnaishi watu wangapi? /***  ***Nyumbani kwako mnaishi watu wangapi?*** |  |
| # Cattle  *SW:* ***Una ng’ombe wangapi?*** |  | # Goats&sheep  *SW:* ***Una mbuzi wangapi?***  ***Una kodnoo wangapi?*** |  | # Donkeys  *SW:* ***Una punda wangapi?*** |  | # Dogs  *SW:* ***Una mbwa wangapi?*** |  | Acres agriculture  *SW:* ***Una shamba? / Ukubwa wa eka ngapi?*** |  |
| # Bicycle  *SW:* ***Una baisikeli?*** |  | # Motorcyle or vehicle  *SW:* ***Una pikipiki au gari?*** */* ***Je, una magari mangapi? Na una pikipiki ngapi?*** |  | # Sofa set  *SW:* ***Una sofa ngapi?*** |  | # Radio  *SW:* ***Una redio ngapi?*** |  | # TV  *SW:* ***Una runinga? / Una televisheni?*** |  |

| Disease  *SW*: ***Magonjwa*** | Have you heard of this disease?^[[1]](#footnote-1)^  *SW:* ***Umeshawahi kusikia kuhusu huu ugonjwa?*** | Do you know if this disease can affect humans or animals^[[2]](#footnote-2)^?  *SW:* ***Huo ugonjwa unaweza kuathiri binadamu au wanyama?***  ***/***  ***Huo ugonjwa unaaambukiza binadamu au wanyama?*** | Do you know what kind of symptoms are caused by this disease?  *Sw:* ***Ni dalili zipi za huo uWgonjwa?*** | Do you know the causes / modes of infection for this disease?  *SW:* ***Mtu anapataje /anaambukizwaje huo ugonjwa?*** | Do you know what kind of treatments are available for this disease?  *SW:* ***Ni matibabu gani ya huo ugonjwa? / Huo ugonjwa unatibiwaje?*** | In case someone is infected by this disease, should this person look for professional help / go to a doctor?  *SW:* ***Ikitokea mtu ana huo ugonjwa aende hospitali au?*** | Do you know how one can prevent getting infected by this disease?  *SW:* ***Unafahamu ni kwa jinsi gani mtu anweza kujikinga na huo ugonjwa?*** | Please rank the three diseases according to their severity / danger (1-3)^[[3]](#footnote-3)^  *SW:*  ***Katika hayo magonjwa matatu, upi ndio unaongoza, upi unafatia na upi ni wa mwisho?***  ***/***  ***Kwenye magonjwa hayo matatu, upi ni namba moja, upi ni namba mbili na upi ni namba tatu?*** |
| --- | --- | --- | --- | --- | --- | --- | --- | --- |
| Anthrax  *SW:* ***Kimeta*** |  |  |  |  |  |  |  |  |
| Brucellosis  *SW:****Brusela*** |  |  |  |  |  |  |  |  |
| Rabies  *SW:* ***Kichaa cha mbwa*** |  |  |  |  |  |  |  |  |

| Who handles livestock?  *SW:* ***Ni nani anahudumia mifugo?***  ***/***  ***Ni nani anachunga mifugo?*** |  | Do you consume milk? Raw or cooked?  *SW:* ***Unakunywa maziwa mabichi au yaliyochemshwa?*** |  |
| --- | --- | --- | --- |
| Who handles dogs?  *SW:* ***Ni nani anahudumia mbwa?*** |  | Do you consume meat? Raw or cooked?  *SW:* ***Unakula nyama mbichi au iliyochemswa?*** |  |
| Do you think that wildlife is neutral, positive or negative for human and livestock health?  *SW:* ***Unafikiri wanyama pori wana faida yoyote kwa binadamu na mifugo, iwe faida kiasi, nzuri au mbaya?*** | | |  |

1. 0=no; 1=yes; [↑](#footnote-ref-1)
2. 0=no; Humans=humans; if animals, specify species; Can also be humans and animals (specify species then)

   *SW:* ***Sifuri ni hapana, kama ni wanyama ainisha (taja) aina za hao wanyama; inaweza kuwa pia binadamu na wanyama (ainisha aina zao)*** [↑](#footnote-ref-2)
3. This was asked at the end of the interview ***(****SW:* ***hiyo iliuzwa mwisho wa mahojiano)*** [↑](#footnote-ref-3)
