# Supplementary figures and images for: Comparative knowledge, attitudes, and practices regarding anthrax, brucellosis, and rabies in three districts of northern Tanzania
Source: BMC Public Health. 2019 Dec 3;19:1625. doi: 10.1186/s12889-019-7900-0 (PMC6889212; doi:10.1186/s12889-019-7900-0)

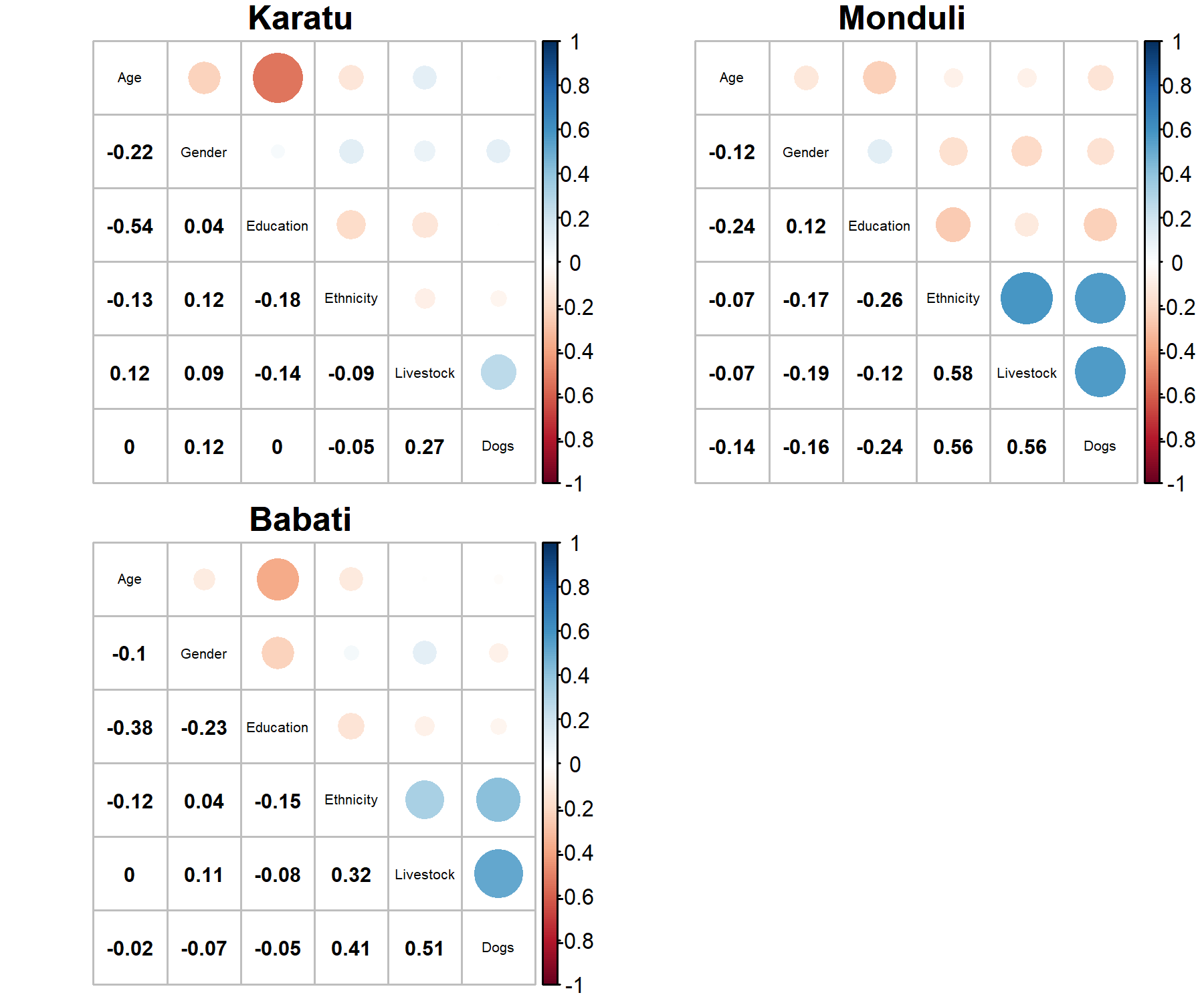

Supplement: Supplementary file 2 — Additional file 2: Figure S2. Pairwise Spearman rank correlation coefficients across explanatory variables. The variable “Dog ownership” (Dogs) was only used in the rabies model, whereas livestock ownership (Livestock) was used in anthrax, and brucellosis models. [file 12889_2019_7900_MOESM2_ESM.tiff]
